# Toxicological Evaluation of Essential Oils from Some Plants of Rutaceae Family

**DOI:** 10.1155/2018/4394687

**Published:** 2018-05-06

**Authors:** Iram Liaqat, Naila Riaz, Qurat-ul-Ain Saleem, Hafiz Muhammad Tahir, Muhammad Arshad, Najma Arshad

**Affiliations:** ^1^Department of Zoology, Government College University, Lahore, Pakistan; ^2^Department of Zoology, University of the Punjab, Quaid-e-Azam Campus, Lahore, Pakistan; ^3^Department of Zoology, University of Education, Lahore, Pakistan

## Abstract

Essential oils are produced as secondary metabolites by aromatic plants, predominantly belonging to families Apiaceae, Lamiaceae, Myrtaceae, and Rutaceae. The family Rutaceae has great economic importance for its numerous edible fruits and essential oils. In the present study, essential oils of seven plants of family Rutaceae,* Aegle marmelos*,* Murraya koenigii*,* Citrus reticulata *Blanco,* Zanthoxylum armatum*,* Skimmia laureola*,* Murraya paniculata*, and* Boenninghausenia albiflora*, were used for their toxicological assessment. Seven groups of selected essential oils-treated Wistar rats were established against control group (*n* = 5) that received water for 14 days; animals were offered feed and water ad libitum and treated with essential oils at 400 mg/kg body weight. Hematological studies revealed significant elevation in TEC in animals treated with essential oils of* M. koenigii*,* S. laureola*, and* B. albiflora*, while an elevation in PCV and depletion in MCV were observed in animals treated with* M. paniculata* and* B. albiflora*, respectively. Serological investigations demonstrated significant depletion in triglycerides and elevation in blood sodium level in animals treated with essential oils of* A. marmelos* and* C. reticulata* Blanco*. Boenninghausenia albiflora* affected many markers including RBC, MCV, triglycerides, HDL, LDL, urea, and sodium. In conclusion, all oils except* B. albiflora* can be considered safe for internal use.

## 1. Introduction

Medicinal plants have been used in traditional treatments for numerous human diseases for thousands of years, particularly in rural areas of developing countries [1]. About 80% of people in these countries use traditional medicines for healthcare purposes [2]. The natural products obtained from medicinal plants are abundant source of biologically active compounds; many of these products are being used in the development of new products for pharmaceuticals and agriculture.

Essential oils are formed by aromatic plants as secondary metabolites and are volatile, natural, complex compounds characterized by a strong odor. These oils were mostly used for their medicinal properties like antibactericidal, antiviral, antifungal, antioxidants, anti-inflammatory, anticancer, antihistamine, and antidiabetic activities and were also used in preservation of foods. All these characteristics have not much changed up to the present day, except that today their mechanism of antimicrobial action is well understood [3].

Rutaceae is a family of flowering plants and is commonly known as the citrus family and is placed in the order Sapindales. The flowers of the species are divided into four or five parts and have strong scents. They range in form and size from herbs to shrubs and small trees. The family is cosmopolitan and contains 154 genera. Some members of the family are plants with highly fragrant flowers and are used in commercial oil production. Some constituents of essential oils, such as citronella and bergamot, are obtained by distillation from plants of this family [4].

Inappropriate use of essential oils can leave adverse effects on human such as skin irritation, headache, and nausea. Caution is generally required if essential oils are to be taken internally or used on food commodities because of the possible cancer-causing effects of some of them [5]. Applied at nonrecommended doses, essential oils can cause functional damage to organs such as stomach and liver in animal and probably in human [6].

In the following study, we focused on* in vitro* antimicrobial toxicity and* in vivo* toxicological aspects of essential oils. For this purpose, an experiment was conducted to check the toxicity of essential oils on Wistar rats. Seven different essential oils of the family Rutaceae were selected:* Aegle marmelos* (bael),* Murraya koenigii* (curry plant),* Citrus reticulata* Blanco (honey plant),* Zanthoxylum armatum* (timber),* Skimmia laureola* (Skimmia),* Murraya paniculata* (Marwa), and* Boenninghausenia albiflora* (Pissu Mar).

## 2. Materials and Methods

The plants were collected from different hilly areas of Pakistan. Essential oils from leaves and stems of all plants were extracted by hydrodistillation method. In this method, the plant material is almost entirely covered with water as suspension and is placed on a burner. Water is made to boil and essential oil is carried over to the condenser along with the steam. Oil forms a layer on water, from where water is withdrawn and oil is collected. The distillation period can take from 15 to 30 minutes or longer.

### 2.1. *In Vitro* Toxicity of Essential Oils

#### 2.1.1. Antimicrobial Assay

The antimicrobial activity of the selected essential oils was checked against two Gram-positive (*Staphylococcus aureus* and* Staphylococcus epidermidis*) and two Gram-negative (*Escherichia coli* and* Klebsiella pneumoniae*) bacterial isolates of clinical importance using disc diffusion and microdilution assays and minimum inhibitory concentration (MIC) and minimum bactericidal concentration (MBC) were determined.

#### 2.1.2. Disc Diffusion Method

Sterilized cotton swabs dipped in respective cultures were swabbed on solidified agar surface. Presterilized filter paper discs with a diameter of 6 mm were placed on the swabbed agar plate. With the help of a micropipette, 5 *μ*l of the respective oil was placed on the disc and plates were incubated for 24 hours at 37°C. At the end of the incubation period, the diameter of the inhibition zone formed around the disc was measured in mm.

#### 2.1.3. Determination of Minimum Inhibitory Concentration (MIC) and Minimum Bactericidal Concentration (MBC) by Microdilution Method

Serial dilutions of essential oils in nutrient broth were prepared and 100 *μ*l of bacterial inoculum was added. Plates were incubated for 24 hours at 37°C. The concentration of oil which leads to absence of visible growth of target strain was declared as MIC, while for MBC, material from the wells showing no growth was subcultured and concentration that caused complete elimination of live bacteria was declared as MBC.

### 2.2. *In Vivo* Toxicological Assessment of Essential Oils

#### 2.2.1. Experimental Design

The experiment was conducted on 24 healthy Wistar rats* (Rattus norvegicus)* purchased from University of Veterinary and Animal Sciences (UVAS), Lahore, weighing 150 grams, and they were kept separately in 12′′ × 18′′ iron cages in the animal house of Zoology Department of University of the Punjab, Lahore, at room temperature and 12-hour day and light period. Chick feed #3 (Hi-Tech, Pakistan) was purchased from local market and animals were provided with feed and water ad libitum. Animals were acclimatized for a period of 7 days before starting the experiment.

#### 2.2.2. Animal Grouping

After acclimatization, animals were divided randomly into eight groups (i.e., I to VIII). Each group was composed of 5 animals. Group I was maintained as control and was given water. Essential oils from* Aegle marmelos, Murraya koenigii, Citrus reticulata *Blanco*, Zanthoxylum armatum, Skimmia laureola, Murraya paniculata*, and* Boenninghausenia albiflora* plants were given to animals from group II to group VIII, respectively.

#### 2.2.3. Dose Preparation

Tween 20 was used for dissolving oils. Each oil was separately mixed in tween 20 in a ratio of 1 : 1 v/v in a vial and labeled. Animals in groups II to VIII were given oral dose of the respective oil by using feeding needle and the treatment was continued for 14 days. A pilot experiment was run to investigate maximal tolerated dose (MTD) of essential oils by using 200, 400, and 800 mg/kg to a group of 3 rats each (data not shown).On the basis of behavioral study including no weight loss > 20% as compared to the first weighing procedure (to be performed the day before the administration) and no death, the volume of the dose given was 400 mg/kg body weight in this study. At the end of the experiment, following 24 hours of last dose, blood samples were collected from all animals one by one. For this purpose, animals were anesthetized by chloroform and blood was drawn directly from the heart with the help of disposable, sterilized plastic syringes (3 cc). A total of 0.5 ml of this blood was placed in EDTA coated vials (B2B, Pakistan) for hematological study. Rest of the blood was kept in refrigerator for 1 hour and centrifuged at 3000 rpm (revolution per minute) for 15 minutes and serum was separated in the clean vial and stored at −20°C.

#### 2.2.4. Hematological Study

The hemoglobin (Hb) contents of blood were estimated by using Randox kit, while total erythrocyte count (TEC) and total leukocyte count (TLC) were measured using automated hematological analyzer following user manual. The packed cell volume (PCV) was measured by microhematocrit method. These hematological values were used for calculation of hematological indices which included MCV, MCH, and MCHC.

#### 2.2.5. Biochemical Parameters

Biochemical parameters were divided into four categories: liver function test (LFT), renal function test (RFT), lipid profile, and electrolytes. LFT included bilirubin, alanine aminotransferase (ALT), aspartate aminotransferase (AST), and alkaline phosphatase (ALP) and RFT included blood urea and creatinine. LFT and RFT were measured photometrically by using diagnostic kits for DiaSys diagnostic system. Lipid profile included total cholesterol, triglycerides, high-density lipoproteins (HDL), and low-density lipoproteins (LDL). All these parameters were measured using kits provided by Beckman diagnostics on automatic analyzer (Beckman Coulter). Electrolytes included blood sodium and potassium level which were measured by flame photometric method.

### 2.3. Statistical Analysis

The values of oil-treated groups were compared with control by using SPSS software. The data was expressed as mean ± SEM (standard error of mean). Statistical comparison was made for each oil with negative control by using independent sample* t*-test. The results were considered as statistically significant when *p* ≤ 0.05.

## 3. Results

### 3.1. *In Vitro* Antimicrobial Activity

#### 3.1.1. Disc Diffusion Assay

The antibacterial activity of seven selected essential oils against four bacterial species is summarized in Table 1. The results of the disc diffusion method revealed that the selected essential oils exhibit varying magnitudes of antibacterial activity. Almost all of the essential oils were active against the selected isolates. According to results,* A. marmelos* had the highest antimicrobial activity against* S. aureus, E. coli*, and* K. pneumoniae*, while* Boenninghausenia albiflora* expressed highest activity against* S. epidermidis*.

#### 3.1.2. Microdilution Assay

Results of MIC and MBC revealed that all the selected essential oils possess antimicrobial activity against two Gram-positive (*S. aureus* and* S. epidermidis*) and two Gram-negative (*E. coli* and* K. pneumoniae*) bacterial isolates. All the oils were found to be more active against Gram-positive isolates as compared to Gram-negative isolates.* Zanthoxylum armatum* and* Murraya paniculata* were the most active essential oils and had smallest MIC;* Citrus reticulata* had least antimicrobial activity. Among all the tested strains,* S. epidermidis* was the most sensitive strain followed by* S. aureus *and* K. pneumoniae*, while* E. coli* was the most resistant strain (Table 2).

### 3.2. Toxicological Evaluation

#### 3.2.1. Hematological Analysis

**Figure 1:**
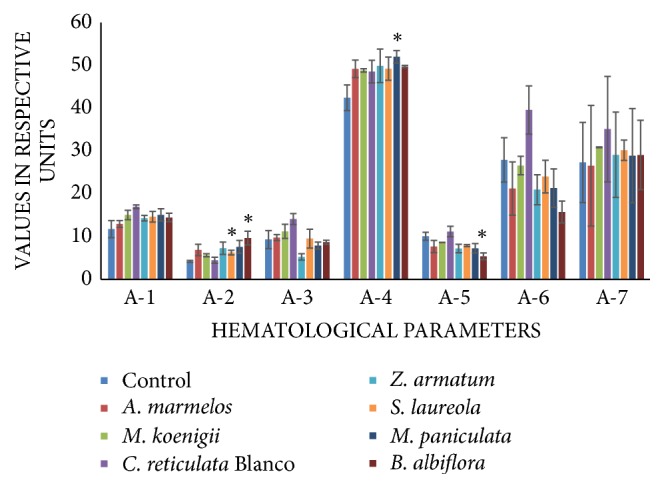
Comparison of hematological parameters in the essential oil- (EO-) fed animals and non-EO-treated (control) animals (I). A-1, hemoglobin (mg/dl); A-2, total erythrocyte count (×10^6^/*μ*l); A-3, total leucocyte count (×10^3^/*μ*l); A-4, packed cell volume (%); A-5, mean corpuscular volume (fL*∗*10/cell); A-6, mean corpuscular hemoglobin (pg/cell); and A-7, mean corpuscular hemoglobin concentration (g/dl). Data is presented as mean ± SEM. Asterisks show significant difference from control.

Hematological studies revealed a significant elevation in TEC in groups VI and VIII. The elevation was 48% and 128%, respectively. PCV was elevated by 22.55% in group VII. MCV decreased by 46.42% in group VIII. There was no alteration in TLC, MCH, and MCHC level in any treated group (Figure 1).

#### 3.2.2. Liver Function Test and Renal Function Test

**Figure 2:**
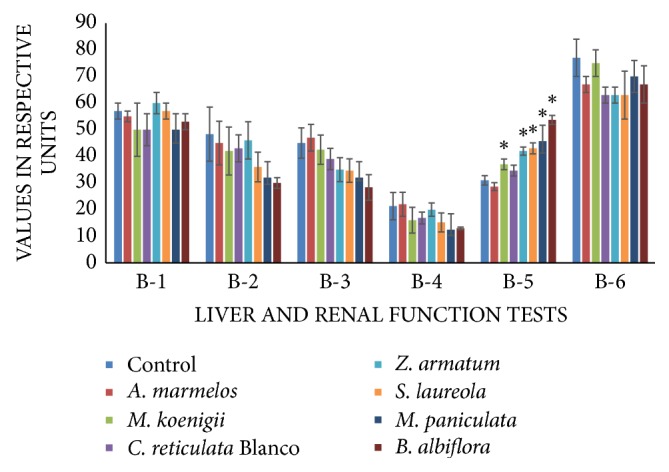
Comparison of liver and renal function tests of essential oil- (EO-) fed animals and control animals. B-1, bilirubin (×100 mg/dl); B-2, alanine transaminase (IU/L) and aspartate transaminase (IU/L); B-3, alkaline phosphatase (U/dl); B-4, urea (mg/dl); and B-5, creatinine (×100 mg/dl). Data are presented as mean ± SEM. Asterisks on respective groups show significant difference from control group at *p* ≤ 0.05.

There was a nonsignificant decrease in bilirubin, ALT, AST, and ALP levels, which are markers for liver injury. An elevated level of blood urea was observed in groups V, VI, VII, and VIII with 25.80%, 38.70%, 47.31%, and 73.11% increase, respectively. Creatinine level remained unchanged in all treatment groups (Figure 2).

#### 3.2.3. Lipid Profile

**Figure 3:**
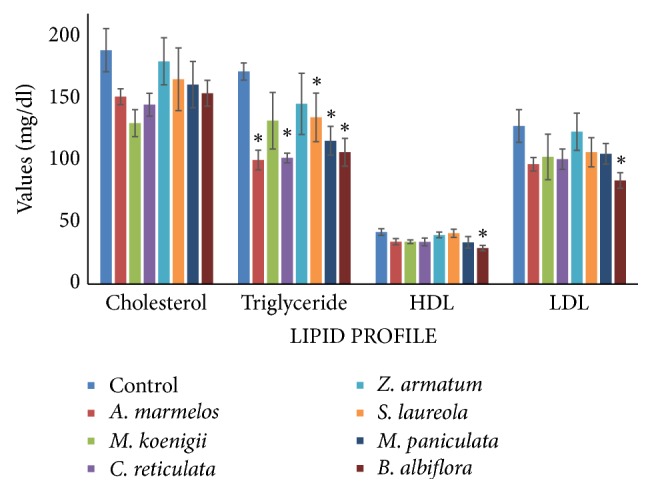
Comparison of lipid profile (mg/dL) in essential oil- (EO-) fed animals and non-EO-treated control animals (I). Data are presented as mean ± SEM, while asterisks on respective EO groups show significant difference from control at *p* ≤ 0.05.

Statistical analysis revealed a significant depletion in triglycerides in groups II, IV, VI, VII, and VIII. The level increased by 49.33%, 48.49%, 31.93%, 41.47%, and 46.15%, respectively. HDL and LDL levels significantly decreased in group VIII by 30.70% and 30.45%, respectively (Figure 3).

#### 3.2.4. Electrolytes

**Figure 4:**
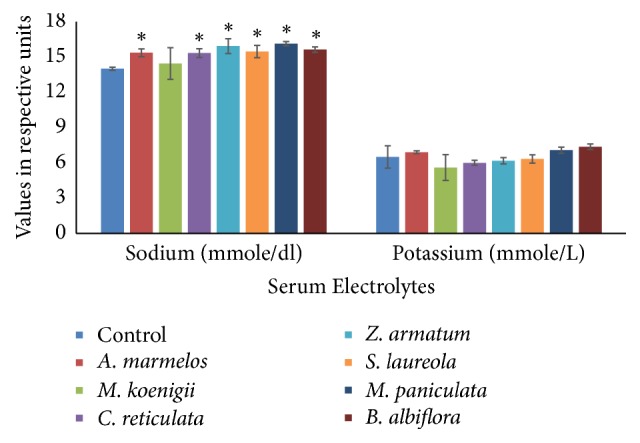
Comparison of blood electrolytes in essential oil- (EO-) fed animals and control animals (I). Data are presented as mean ± SEM. Asterisks on respective EO groups show significant difference from control at *p* ≤ 0.05.

The results revealed that all the selected oils have potential to elevate blood sodium level. Statistical analysis indicated a significant difference in groups II, IV, V, VI, VII, and VIII. The increase in level was 11.89%, 11.65%, 16%, 12.62%, 17.47%, and 13.83% respectively. There was no alteration observed in blood potassium level in any essential oil fed group (Figure 4).

## 4. Discussion

The increased use of antimicrobial agents has resulted in the development of resistant strains of bacteria. So there is a need to develop effective antimicrobial agents with new modes of action against these pathogenic microbes. Essential oils have been recognized as a rich source of new bioactive secondary metabolites, which possess the potential of treatment of many infectious diseases. In the current study, the potential of selected essential oils as antimicrobial agents was screened against four resistant clinical isolates including two Gram-positive (*S. aureus* and* S. epidermidis*) and two Gram-negative (*E. coli* and* K. pneumoniae*) bacteria. The antibacterial activity was initially screened by using disc diffusion method and then the minimum inhibitory concentration (MIC) and minimum bactericidal concentration (MBC) were determined by using microdilution method.

The results of the disc diffusion method revealed that the selected essential oils exhibit varying magnitudes of antibacterial activity. Almost all of the essential oils were active against the four clinical isolates. According to results,* Aegle marmelos* was found to possess the highest antimicrobial activity against* S. aureus, E. coli*, and* K. pneumoniae*, while* Boenninghausenia albiflora* shows highest activity against* S. epidermidis*. All the oils were found to be more active against Gram-positive bacterial isolates (*S. aureus* and* S. epidermidis*) and were less active against Gram-negative bacteria (*E. coli *and* K. pneumoniae*) [7].

Results of MIC and MBC also revealed that all the oils were more active against Gram-positive as compared to Gram-negative bacterial isolates.* Zanthoxylum armatum* and* Murraya paniculata* were the most active essential oils and were active against all the selected isolates.* Citrus reticulata* Blanco was the least active oil with low antibacterial activity. Gram-negative bacteria are more resistant to essential oils then Gram-positive bacteria due to permeability barrier provided by extra lipopolysaccharide membrane. The antimicrobial activity of essential oils could be due to their hydrophobic characteristic, due to which these oils are capable of breaking the lipids of bacterial cell membrane and making them more permeable [8].

The current study involved the investigation of hematological and serological changes in rats fed with essential oils. The hematological parameters can be influenced by toxicity of essential oils as these oils have ability to initiate the acute phase response [9]. Significant difference was observed in RBC, PCV, and MCV levels between the treated and control groups. However, in most of the oil feeding groups, the Hb, TEC, TLC, PCV, MCV, MCH, and MCHC were in normal range.

Hemoglobin is a metalloprotein that contains iron and is present in red blood cells of all vertebrates. It transports oxygen from respiratory organs to the rest of the body. In the current study, the results indicate that the hemoglobin level nonsignificantly increased in oil-fed groups. The reason for this slight increase in hemoglobin was an increase in the number of red blood cells. Erythrocytes are the most common type of blood cells and are the main source of transferring oxygen to the body tissues. RBCs are formed in bone marrow and stimulated by decrease in oxygen which is detected by kidneys [10]. In the current study, a significant elevation in TEC was noticed in the groups of animals, which were treated with* M. koenigii*,* S. laureola*, and* B. albiflora* essential oils. Studies had shown that morphological changes occur in RBCs in response to various treatments by toxic agents [11]. According to Suwalsky et al. [12],* Balbisia peduncularis* extract caused changes in the normal erythrocyte morphology. An elevation in total erythrocytes was noticed in some EO-treated groups. Packed cell volume (PCV) or hematocrit is the volume percentage of red blood cells in blood. PCV was slightly increased in all essential oil-fed groups but a significant elevation was observed only in group treated with essential oil of* M. paniculata*. This increase in PCV may be due to an increase in RBCs number in the oil-fed groups [13]. This finding is supported by an elevated erythrocytes number in the same group. Mean corpuscular volume (MCV) is a measure of the average red blood cell size. The results indicated significant decrease in the level of MCV in the group treated with essential oil of* B. albiflora*. This reduction in MCV was maybe due to decrease in size of red blood cell [13]. There was no significant difference observed in MCH and MCHC in any oil-fed group. That was because of the hemoglobin level which was in normal range in all groups.

Leukocytes are involved in the defense mechanism of the body against foreign materials. In the present study, no significant change was observed in any oil-fed group when compared to control group. All the groups indicate the number of white blood cells in normal range. This is the indication that the selected essential oils perhaps do not affect leucopoiesis or half-life of leukocytes.

The serological parameters selected for the study included the liver function test, renal function test, lipid profile, and serum electrolytes. The selected essential oils cause a nonsignificant depletion in the level of all the markers for liver injury, which included bilirubin, alanine aminotransferase (ALT), aspartate aminotransferase (AST), and alkaline Phosphatase (ALP). This is an indication that all these oils do not have toxic effects particularly in reference to liver function test.

Renal function test is an indication of the state of kidney. Urea and creatinine are usually considered as markers with respect to kidney function. Urea is the nitrogenous waste product of the body. Raised level of blood urea is an indication of renal dysfunction. An elevated level of blood urea was observed in some of the essential oil-fed groups. Significant elevation was observed in groups treated with* Z. armatum*,* S. laureola*,* M. paniculata,* and* B. albiflora* essential oils. This increase in urea level may be due to epithelial necrosis to the renal tubules [14]. On the other hand, blood creatinine level remained unaltered in all essential oi-fed groups. Indicating EO at this dose may cause partial toxicity to kidneys. According to Abarikwu et al. [15], whenever the urea level increased and creatinine level is reduced, there is no kidney damage. Nisha et al. [16] reported that renal toxicity should be considered only when creatinine and urea level increased parallel to each other.

Lipid profile is the term given to the evaluation of total cholesterol, triglycerides, high-density lipoproteins (HDL), and low-density lipoproteins (LDL). This test is mostly used to identify hyperlipidemia, which is risk factor for heart diseases. Cholesterol is an ester and is an important part of mammalian cell membrane [17]. It is formed in the liver. In this study, no variation was observed in cholesterol level in any essential oil-fed group. According to Rajadurai and Prince [18], the cholesterol level slightly decreases in rats in treatment with* Aegle marmelos* leaf extracts.* Murraya koenigii* leaf extracts also decrease cholesterol level in rats [19].

Triglyceride is also an ester and is derived from glycerol and three fatty acids. High triglyceride is directly related to coronary heart disease. Our results indicate a decrease in triglycerides level in all essential oil-fed groups as compared to control group. Statistical analysis indicated a significant depletion in groups treated with* A. marmelos, C. reticulata *Blanco*, S. laureola, M. paniculata*, and* B. albiflora*. Our findings are in accordance with Rajadurai et al. [18] and Rajadurai et al. [19]; they reported that the leaf extracts of* A. marmelos* cause a decrease in triglycerides level in rat models.

Recent studies suggest that triglyceride itself is independently related to coronary heart disease [20, 21] and most of the antihypercholesterolemic drugs do not decrease triglycerides levels but plant extracts do. These findings indicate beneficial effects of essential oils instead of their toxicological potential

High-density lipoprotein (HDL) is also known as good cholesterol. It transports other lipids like cholesterol and triglycerides in the blood stream. Various studies have shown that an increase in HDL-cholesterol is associated with a decrease in coronary risk [22]. Statistical analysis indicated a significant decrease in HDL in animals treated with* B. albiflora* essential oil. So this essential oil may be considered as toxic because it is decreasing good cholesterol.

Low-density lipoproteins (LDL) are also known as bad cholesterol. LDL collects in the walls of blood vessels and causes the blockage of the arteries. Higher LDL levels may cause sudden blood clot in an artery and increase risk of heart attack. In present study, significant difference was observed in animals treated with* B. albiflora*. Our findings are in accordance with Kesari et al. [19] who reported that the administration of leaf extracts of* M. koenigii* causes a decrease in the level of LDL of normal and diabetic rats. So these essential oils play a beneficiary role.

Sodium and potassium are electrolytes present in blood and other body fluids. These help keep the water and electrolyte balance of the body and are also important in the proper functioning of nerves and muscles. The hormone aldosterone controls the level of sodium and potassium in the body. The results revealed that all the selected essential oils have potential to elevate blood sodium level. Statistical analysis indicated a significant elevation in animals treated with* A. marmelos, C. reticulata *Blanco*, S. laureola, M. paniculata*, and* B. albiflora*. Similar findings have been reported by Odeyemi et al. [23] who concluded that all essential oils may not be safe as these can lead to leakage of electrolytes from the cells. However, in current study, blood potassium level remained unaltered.

In conclusion, although EOs were found to have some favorable insinuations (decrease in triglycerides and LDL along with no adverse effects on LFT and RFT), they may leave specific undesired effects (e.g., increase in urea* (Z. armatum)*, depletion in HDL* (B. albiflora)*, induction in erythropoiesis above normal level (*M. koenigii*,* S. laureola*, and* B. albiflora*), and increase in sodium (*A. marmelos, C. reticulata* Blanco,* S. laureola*,* M. paniculata*, and* B. albiflora*)), which point to their toxic potential.

## 5. Conclusion

As reduction in triglycerides is considered as beneficial and change in urea without alteration in creatinine is not considered as toxic output of any tested material, it could be concluded that all oils except* B. albiflora* can be considered safe for internal use with caution and sodium level may be continuously monitored.* B. albiflora *affected many markers including RBC, MCV, triglycerides, HDL, LDL, urea, and sodium.

## Figures and Tables

**Table 1:** Zone of inhibition of different essential oils against four bacterial isolates.

Oils	Gram-positive bacteria		Gram-negative bacteria	
	*S. aureus *(mm)	*S. epidermidis *(mm)	*E. coli *(mm)	*K. pneumonia *(mm)
I	24	18	18	20
II	10	14	10	14
III	16	12	11	10
IV	14	15	11	10
V	18	18	13	14
VI	14	15	9	12
VII	18	20	8	15

Zone of inhibition of selected essential oils against *S. aureus, S. epidermidis, E. coli, *and* K. pneumoniae *(I, *Aegle marmelos*; II, *Murraya koenigii*; III, *Citrus reticulata *Blanco; IV, *Zanthoxylum armatum*; V, *Skimmia laureola*; VI, *Murraya paniculata*; and VII, *Boenninghausenia albiflora*).

**Table 2:** Minimum inhibitory concentration (MIC) and minimum bactericidal concentration (MBC) of different oils against four bacterial isolates (% v/v).

Essential oils	Gram-positive bacteria				Gram-negative bacteria			
	*S. aureus*		*S. epidermidis*		*E. coli*		*K. pneumoniae*	
	MIC	MBC	MIC	MBC	MIC	MBC	MIC	MBC
*Aegle marmelos*	5	10	5	5	5	10	2.5	2.5
*Murraya koenigii*	2.5	2.5	2.5	5	5	5	2.5	2.5
*Citrus reticulata *Blanco	5	5	2.5	5	10	20	5	10
*Zanthoxylum armatum*	0.312	0.63	1.25	2.5	5	5	2.5	2.5
*Skimmia laureola*	0.625	0.63	1.25	1.25	5	5	5	5
*Murraya paniculata*	1.25	2.5	1.25	2.5	2.5	2.5	5	5
*Boenninghausenia albiflora*	2.5	2.5	0.156	0.16	2.5	5	5	5

## Data Availability

The data used to support the findings of this study are included within the article.
